# Expulsion of Infarcted Myoma Following Ultrasound-Guided Uterine Artery Embolization: A Fertility-Preserving Approach

**DOI:** 10.7759/cureus.31129

**Published:** 2022-11-05

**Authors:** Nidhi Goyal, Manjusha Agrawal, Manila Eleti

**Affiliations:** 1 Department of Obstetrics and Gynaecology, Jawaharlal Nehru Medical College, Datta Meghe Institute of Medical Sciences, Wardha, IND

**Keywords:** uterus, leiomyoma, fertility preserving, menorrhagia, uterine artery embolization, infarcted myoma

## Abstract

The most frequent benign tumor of the female pelvis, uterine fibroids (leiomyomas), have a lifetime frequency of about 70% among Caucasian women. The most preferred treatment for fibroids is still hysterectomy, albeit there are issues with its misuse. Today, uterine artery embolization (UAE) is a well-recognized minimally invasive treatment for symptomatic fibroids. A 29-year-old female came with heavy menstrual bleeding for two months. Ultrasonography revealed a large fibroid of 8cm x 7cm in the posterior wall of the myometrium. She underwent uterine artery embolization for the same. The fibroid was expelled through the vagina in small portions over one month following the intervention. There was a significant reduction in the fibroid size with a considerable amount of symptomatic relief to the patient within a month. The most prevalent benign pelvic tumor, uterine fibroids, affects over 40% of women of reproductive age. Uterine artery embolization is a safe and successful alternative to surgery for treating symptomatic fibroids, with significantly lower morbidity and mortality. It also preserves fertility, giving the patient hope for a future pregnancy.

## Introduction

Benign tumors of the uterus are known as uterine fibroids or leiomyomas [1]. With an accumulative incidence of well over 40% by the age of 50, they are quite prevailing. Most of the women are asymptomatic; however, 20% may present with symptoms that are either abnormal uterine bleeding or pressure symptoms like bladder and bowel dysfunction, abdominal protrusion, dysmenorrhea, and infertility [2]. The most common option for treating a symptomatic uterine fibroid has been a hysterectomy. Hysterectomy, however, comes with its own set of risks and issues, including early surgical menopause and infertility [3]. Alternatives to hysterectomy include the use of NSAIDs, oral contraceptive pills, levonorgestrel IUD, GnRH agonists, GnRH antagonists, and endometrial ablation. In the last few decades, in addition to medical treatment, several semi-invasive and uterus-preserving operations have been developed in the quest to reduce morbidity [4]. Uterine artery embolization (UAE), recommended for premenopausal women who failed hormonal control and want to keep the uterus, is a treatment option for uterine fibroids to reduce abnormal bleeding and pain/pressure symptoms [2]. UAE blocks off the fibroid's blood supply, causing it to undergo necrosis and shrink in size.

## Case presentation

A 29-year-old female with Para 1 Living 1 with a previous cesarean section came to the outpatient department with complaints of menorrhagia and dysmenorrhea for two months. Symptoms were not relieved on medical intervention (OC pills and tranexamic acid). Bleeding was irregular, lasted for 15 to 20 days every month, associated with the passage of clots and dysmenorrhea. She was averagely built (BMI of 22kg/m2). She was a known case of hypothyroidism, ischemic heart disease, and hypertension and was on tablet met XL 50 mg once a day and tablet telmisartan 40 mg once daily. Her blood pressure was 144/92 mmHg, and her pulse was 64 beats per minute. Per abdomen was soft, with no palpable mass. Cervix was pulled up with excessive white discharge per speculum examination. Her complete blood count, coagulation profile, and thyroid profile were within normal limits.

USG revealed a large submucosal fibroid of 8cm x 7cm in the posterior wall of the myometrium and had moderate vascularity on doppler. The patient underwent uterine artery embolization by an intervention radiologist for the fibroid. A fluoroscopy guided coaxial 2-3 French catheter was introduced into the uterine artery via the femoral artery, and polyvinyl alcohol (PVA) was injected. Bilateral arteries were embolized, which went uneventful was discharged after explaining all the possible postoperative complications associated with the procedure and was advised of a weekly follow-up.

After five days, the patient returned to the hospital with complaints of something coming out of the vagina not associated with pain. On examination, a smooth, soft, brownish-red colored, malodours 5cm x 5cm tongue-shaped mass was seen coming out, occupying the upper vagina. Uterus and External OS could not be palpated. She was hospitalized and started on empirical IV antibiotics to avoid sepsis.

**Figure 1:**
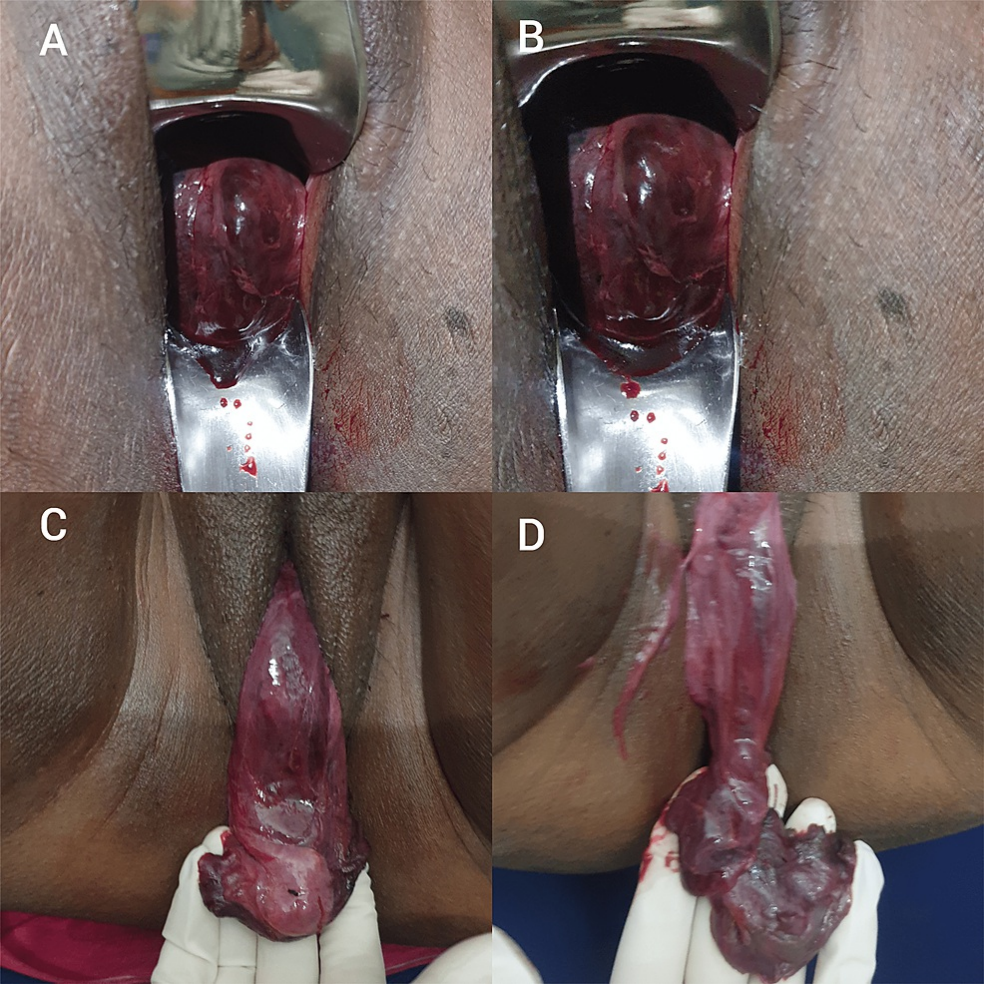
Shows gradual descent and expulsion of infarcted myoma piecemeal from A to D through the vagina over a few days.

USG revealed that there was prolapse of fibroid into the cervical canal, which has undergone necrosis. The uterus was normal in position. It also revealed that the fibroid has shrunken in size, and no abnormal vasculature of the fibroid could be seen. The mass descended more within a few days until it spontaneously expelled from the vagina (Figure 1). Mass was blackish red, smooth, soft, and foul smelling. It was expelled through the vagina piecemeal for the next two months. Prolapsed tissue sent for histopathology was suggestive of leiomyoma.

**Figure 2:**
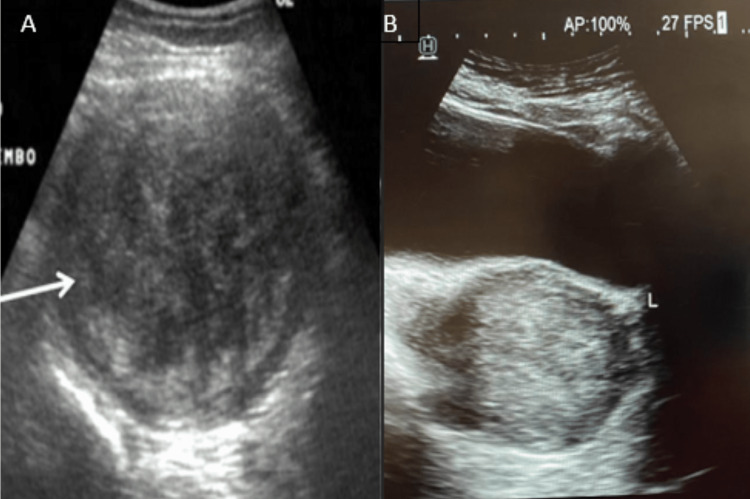
Shows USG comparing the size of fibroid pre-embolization (8cmx7cm) (Figure A) and post-embolization (5cmx4cm) (Figure B) USG: Ultrasonography

After three months, sonography revealed an almost completely regressed fibroid (Figure 2). Post-procedure there was a significant decrease in her symptoms like menorrhagia and dysmenorrhea. 

## Discussion

Our study reports a case of a young female with large sized intramural posterior wall fibroid. As the surgery was relatively contraindicated pertaining to her medical condition, uterine artery embolization in association with the interventional radiology department was done. In this case, the fibroid mass was expelled piecemeal through the vagina via the cervical canal from the uterus in about six weeks. Blood loss (1 pad/ day soakage) and post-procedural pain were minimal, and the patient’s symptoms were relieved. The hospital stay was less than three days.

In women of reproductive age, a uterine fibroid is a common source of upsetting symptoms. Several therapeutic modalities, such as myomectomy (abdominal/vaginal/hysteroscopic), myolysis, endometrial ablation, uterine artery occlusion, uterine artery embolization, medical therapy with gonadotrophin-releasing hormone agonist, progestational compounds, etc., are uterine fibroid options that preserve the uterus. Focused high-frequency ultrasound therapy with MRI guidance has also been tried recently [4].

After uterine artery fibroid embolization, there is a 50 % reduction in fibroid size and a 90% reduction in pressure symptoms which has been substantiated by many other studies [1,5-7].

Although uterine artery embolization is an effective modality to treat symptomatic fibroids, it also has some limitations. Expulsion of fibroid tissue in the form of small tissue fragments via the vagina is a minor complication and is mostly uneventful. Although, complications can occur when a partially exposed piece of an infarcted fibroid persists through the cervix, forming a nidus for an infection that may cause endometritis. Patients presenting with this problem require admission and immediate supportive care, including intravenous fluids and antibiotics to avoid sepsis which accounts for a risk of 2% [2]. Other complications include uterine ischemia, uterine necrosis, chronic vaginal discharge, ovarian and sexual dysfunction, and treatment failure [8]. The location of the fibroid is a more relevant factor related to complications rather than size. Several studies have shown that fibroids may regrow, especially in younger age groups [7].

## Conclusions

Uterine artery embolization is emerging as an alternative to the conventional treatment of symptomatic uterine fibroids. It has been proven safe and efficient for symptomatic uterine fibroids. Reproductive outcomes after uterine artery embolization are still not very clear, and it still gives a chance for future pregnancies in patients with symptomatic uterine fibroid as compared to other treatment options like hysterectomy.
